# A Mouse Model of Partial Pancreas Agenesis Induced by *Polo‐like kinase 1* Mutation

**DOI:** 10.1096/fj.202501377R

**Published:** 2025-08-13

**Authors:** Xiyue Chen, Zhihao Jia, Shihuan Kuang

**Affiliations:** ^1^ Department of Animal Sciences Purdue University West Lafayette Indiana USA; ^2^ Cambridge‐Suda Genomic Resource Center, Suzhou Medical College Soochow University Suzhou China; ^3^ Department of Orthopedic Surgery and Department of Pathology Duke University School of Medicine Durham North Carolina USA; ^4^ Duke Cancer Institute Durham North Carolina USA

**Keywords:** diabetes mellitus, hyperglycemia, insulin, metabolism, pancreas, polo‐like kinase 1

## Abstract

The pancreas regulates metabolic homeostasis through exocrine and endocrine pathways. Dysfunction or loss of pancreatic β‐cells causes diabetes. Here we explore the role of Polo‐like kinase 1 (PLK1) in the pancreas using a pancreatic‐lineage specific knockout (*Plk1* ^PKO^) mouse model. *Plk1* ^PKO^ leads to partial pancreatic agenesis, diminishing pancreatic mass. Adult *Plk1* ^PKO^ mice exhibit diabetic syndromes including hyperglycemia, glucose intolerance, and insulin hypersensitivity. *Plk1* ^PKO^ mice also exhibit growth retardation and reduced skeletal muscle and adipose tissue masses. Furthermore, *Plk1* ^PKO^ mice develop metabolic adaptation towards fatty acid utilization, manifested by elevated oxygen consumption (VO_2_), reduced respiratory exchange ratio (RER), and more oxidative myofibers. These findings reveal a key role of PLK1 in pancreas development.

## Graphical abstract

These findings demonstrate a critical role of PLK1 in pancreas development. Pancreas‐specific *Plk1* knockout causes severe pancreatic agenesis and impaired insulin secretion in adult mice; consequently, it leads to diabetic syndromes, growth retardation, and systemically metabolic disorder.

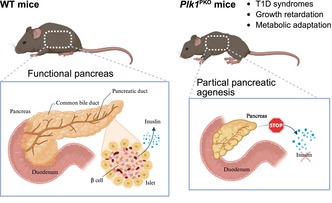

## 1 Introduction

The pancreas arises from progenitors expressing pancreatic and duodenal homeobox 1 (*Pdx1*) [1]. These progenitors are specified into dorsal and ventral buds at around embryonic day E8.5 in mice, and further differentiate into both endocrine and exocrine cells [1]. Partial or complete agenesis of the pancreas has been documented under rare circumstances with unknown etiology [2]. Disruption of pancreatic development or function causes hyperglycemia and diabetes milieus. For example, neonatal diabetes and maturity‐onset diabetes of the young (MODY) result from pancreatic developmental defects [3]. Type 1 and 2 diabetes are caused by autoimmune β‐cell destruction and insulin resistance/β‐cell dysfunction, respectively [3].

PLK1 is a cell cycle regulator overexpressed in pancreatic cancers to promote cell proliferation and migration [4]. PLK1 also regulates normal mitotic cells during development, and its deletion leads to embryonic lethality [5, 6]. In the adult pancreas, PLK1 mediates adaptive proliferation of β‐cells in response to insulin demand under situations such as metabolic stress or obesity [7]. Here, we generated a pancreatic lineage‐specific *Plk1* knockout (*Plk1* ^PKO^) model to examine the role of PLK1 in pancreas development. Our results demonstrate that the *Plk1* ^PKO^ mice fail to form a normal pancreas, leading to diabetes in adult mice.

## 2 Methods

### 2.1 Experimental Animals

All mice procedures were approved by Purdue University Animal Care and Use Committee. *Plk1* ^*flox/flox*^ mice [8] were crossed with *Pdx1*‐Cre mice (Jaxmice#014647). Unless noted, 2–5 months old male and female mice were used.

### 2.2 Blood Glucose and Insulin Measurements

For glucose tolerance tests (GTT), mice were overnight‐fasted and injected i.p. with D‐glucose (2 g/kg body weight). For insulin tolerance tests (ITT), mice were 4 h‐fasted and injected i.p. with insulin (0.75 U/kg body weight). Tail blood glucose was measured using Accu‐Check Active. Serum insulin was measured using ELISA (EMINS, Thermo Scientific).

### 2.3 Indirect Calorimetry Study

VO_2_, CO_2_ production (VCO_2_), RER, and heat production were measured using an indirect calorimetry system under a 12‐h light/dark cycle at 22°C. Data were normalized to lean mass, with means over 12‐h cycles.

### 2.4 H&E Staining

Tissues were fixed with 4% PFA, dehydrated, and paraffin‐embedded. 6‐μm sections were stained with hematoxylin (15 min) and eosin (1 min). Images were captured with a Leica DM6000B microscope.

### 2.5 Immunofluorescence Staining

Tissue sections were fixed in 4% PFA, quenched with 100 nM glycine, and blocked in buffer (5% goat serum, 2% BSA, 0.2% Triton X‐100). Primary antibodies (INSULIN, Santa Cruz, sc‐8033; MIST1, gift from Dr. Stephen Konieczny; type I myosin, DSHB, BA‐D8; type IIA myosin, DSHB, SC‐71) were incubated at 4°C overnight, followed by secondary antibodies and DAPI. For each section, the entire sectional area was assessed to encompass both islet and non‐islet regions.

### 2.6 Treadmill Test

Mice were trained on a treadmill (Eco3/6, Columbus Instruments, Columbus, OH, USA) for 3 consecutive days (10 m/min, 10 min/day). Mice then ran at increasing speeds (10 m/min for 5 min, then at 2 m/min increments every 2 min) until mice were exhausted. Running distance, time, and maximum speed were recorded.

### 2.7 Statistical Analysis

Data are presented as mean ± S.E.M (*n* ≥ 3). Statistical analysis was performed using two‐tailed student's *t*‐test. GraphPad Prism was used to generate graphs. *p* < 0.05 was considered significant.

## 3 Result

### 3.1 *Plk1* ^PKO^ Mice Exhibit Pancreas Agenesis, Glucose Intolerance, and Insulin Hypersensitivity

**FIGURE 1:**
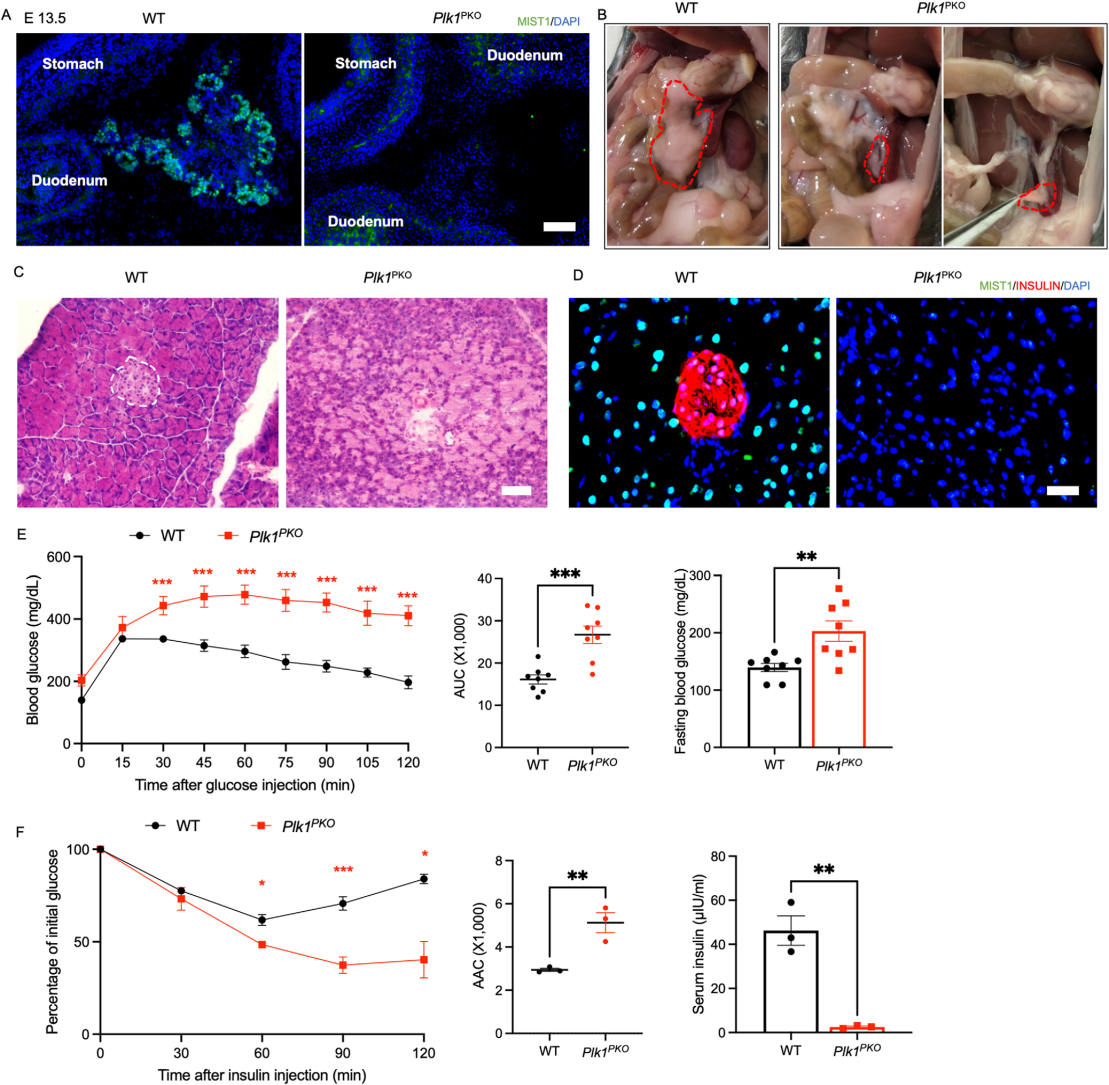
*Plk1* ^PKO^ mice develop pancreas agenesis, leading to glucose intolerance and insulin hypersensitivity in adult mice. (A) MIST1 staining from WT and *Plk1* ^PKO^ mice at E 13.5. DAPI marks nuclei. Scale bar: 50 μm. (B) Representative images of pancreas from 3‐month‐old WT and *Plk1* ^PKO^ mice; pancreas was indicated by red dash line. (C) Representative H&E staining of pancreas from 3‐month‐old WT and *Plk1* ^PKO^ mice. Scale bar: 50 μm. Islet was indicated by white dashed line. (D) MIST1 and INSULIN staining of pancreas from 3‐month‐old WT and *Plk1* ^PKO^ mice. Scale bar: 25 μm. (E) Blood glucose levels (left) and area under the curve (AUC) (middle) during glucose tolerance tests (GTT), and 12 h‐fasting blood glucose levels (right) from 3‐month‐old WT and *Plk1* ^PKO^ mice (*n* = 8 mice per group). (F) Percentage of initial glucose (left) and area above curve (AAC) (middle) during insulin tolerance tests (ITT), and serum insulin levels (right) from 3‐month‐old WT and *Plk1* ^PKO^ mice (*n* = 3 mice per group). * *p* < 0.05, ** *p* < 0.01, *** *p* < 0.001.

WT and *Plk1* ^PKO^ E13.5 embryos were sectioned across the abdominal area (Figure 1A). In WT embryos, pancreatic buds marked by MIST1^+^ acinar progenitors were evident, but similar structures were not observed in *Plk1* ^PKO^ embryos (Figure 1A). Adult (3‐month‐old) *Plk1* ^PKO^ mice exhibited only a fibrous stromal remnant at sites where the pancreas was normally found in WT mice (Figure 1B). Under H&E staining, the remnant contained disorganized structures lacking any islets (Figure 1C). Immunofluorescence staining of MIST1 and INSULIN indicated the absence of both acinar and β‐cells in *Plk1* ^PKO^ (Figure 1D). These results demonstrate that *Plk1* ^PKO^ abolishes embryonic pancreatic development, resulting in pancreatic agenesis.

Adult *Plk1* ^PKO^ mice had significantly lower serum insulin levels and higher fasting blood glucose concentrations than WT mice (Figure 1E,F, right). GTT showed impaired glucose clearance in *Plk1* ^PKO^ mice, with higher blood glucose levels following glucose injection and a larger area under curve (AUC) (Figure 1E, left, middle). ITT revealed enhanced insulin sensitivity in *Plk1* ^PKO^ mice, manifested by faster insulin‐mediated glucose clearance and a larger area above curve (AAC) (Figure 1F, left, middle). These results indicate that *Plk1* ^PKO^ mice exhibit hypoinsulinemia, hyperglycemia, and hypersensitivity to insulin. The anatomical and physiological analyses together demonstrate that PLK1 is essential for pancreas development, and its loss‐of‐function leads to pancreatic agenesis and diabetes in adult mice.

### 3.2 *Plk1* ^PKO^ Mice Exhibit Reduced Survival, Growth Retardation, and Metabolic Abnormalities

**FIGURE 2:**
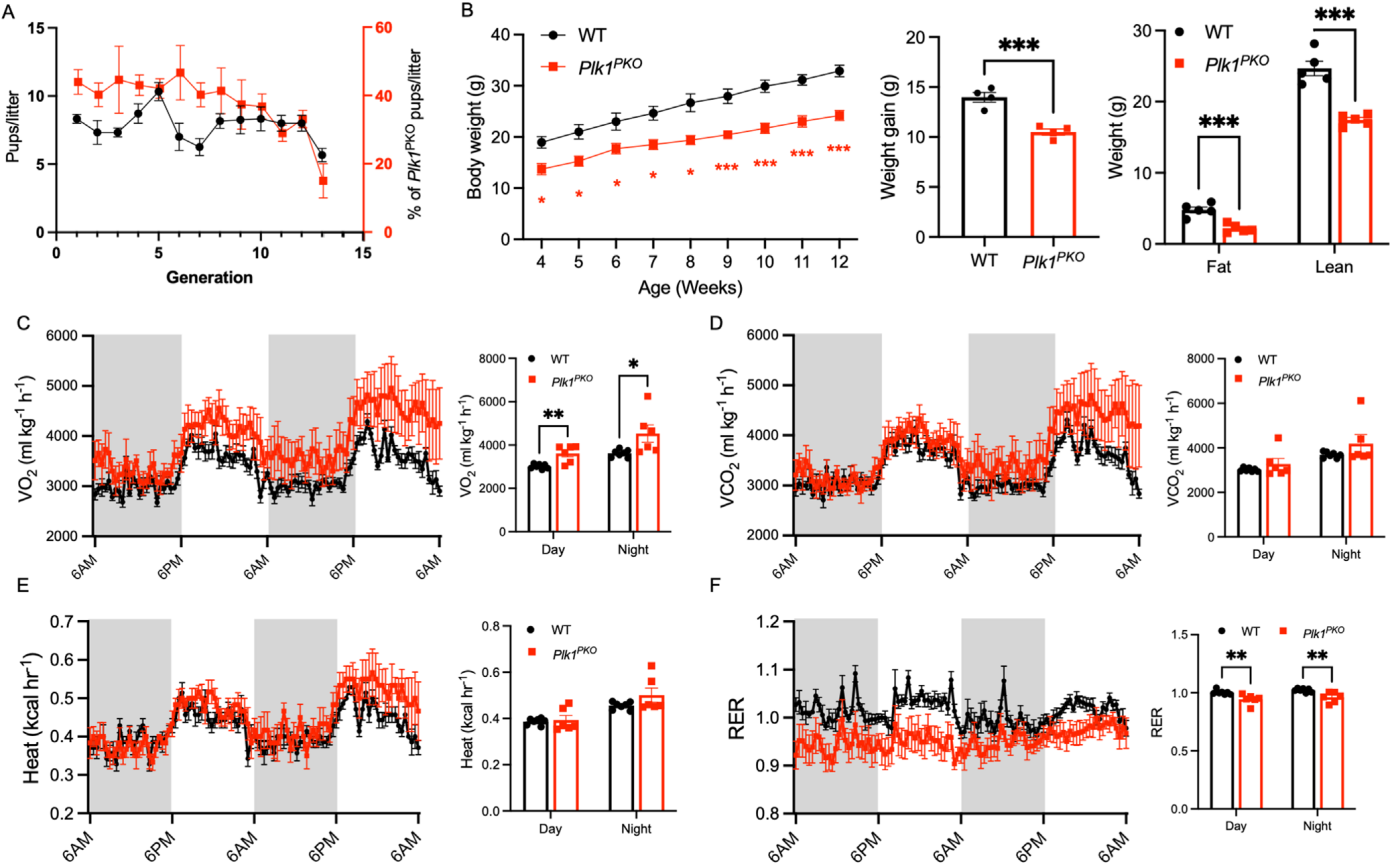
Pancreas‐specific *Plk1* KO attenuates weight and causes metabolic abnormalities. (A) Number of pups and frequency of *Plk1* ^PKO^ pups per litter over 13 generations (at least 3 litters were assessed in each generation, total *n* = 396 pups from 51 litters). (B) Growth curves (left) and weight gain (middle) from 4–12 weeks of age in WT and *Plk1* ^PKO^ mice (*n* = 4 mice per group). Right: Fat and lean mass in 2‐month‐old WT and *Plk1* ^PKO^ mice determined by EcoMRI body composition analyzer (*n* = 5 mice per group). (C) Oxygen consumption rate (VO_2_) (left) and quantification (right) at light and dark based on 2‐month‐old WT and *Plk1* ^PKO^ mice (WT, *n* = 6 mice; *Plk1* ^PKO^, *n* = 7 mice). (D) Carbon dioxide production (VCO_2_) (left) and quantification (right) at light and dark in 2‐month‐old WT and *Plk1* ^PKO^ mice (WT, *n* = 6 mice; *Plk1* ^PKO^, *n* = 7 mice). (E) Heat production (left) and quantification (right) at light and dark in 2‐month‐old WT and *Plk1* ^PKO^ mice (WT, *n* = 6 mice; *Plk1* ^PKO^, *n* = 7 mice). (F) Respiration exchange rate (RER) (left) and quantification (right) at light and dark in 2‐month‐old WT and *Plk1* ^PKO^ mice (WT, *n* = 6 mice; *Plk1* ^PKO^, *n* = 7 mice). * *p* < 0.05, ** *p* < 0.01, *** *p* < 0.001.

We compared birth rates of *Plk1* ^PKO^ mice from *Pdx1‐Cre/Plk1* ^*fl/fl*^ × *Plk* ^*fl/fl*^ breeders. *Plk1* ^PKO^ mice were born slightly under the expected 50% Mendelian ratio, at ~43% (*n* = 256 pups, 32 litters) for the first 8 generations (Figure 2A). The ratios then gradually declined to ~15% by the 13th generation (Figure 2A). Postnatally, *Plk1* ^PKO^ mice were viable but smaller than WT mice at 4 weeks old, with a slower growth rate (Figure 2B, left) and ~30% reduction in weight by 2‐month‐old (Figure 2B, middle). Body composition analysis revealed reductions in both lean and fat masses (Figure 2B, right). These results suggest that pancreas agenesis affects embryonic survival and postnatal growth of mice.

*Plk1* ^PKO^ mice exhibited higher VO_2_ rates than WT mice (Figure 2C). However, VCO_2_ and heat production were similar in *Plk1* ^PKO^ and WT mice (Figure 2D,E). RER was significantly lower in *Plk1* ^PKO^ than in WT mice (Figure 2F), indicating that *Plk1* ^PKO^ increases lipid utilization. These findings demonstrate that *Plk1* ^PKO^ leads to systemic metabolic reprogramming.

### 3.3 *Plk1* ^PKO^ Alters Skeletal Muscle and Adipose Tissue Structure and Function

We further assessed how pancreatic agenesis affects peripheral metabolic organs. Adult *Plk1* ^PKO^ mice had lighter tibialis anterior (TA), quadriceps (QU), gastrocnemius (GAS) and extensor digitorum longus (EDL) muscles, compared to WT mice (Figure S1A). *Plk1* ^PKO^ mice had reduced maximum speed, running time, and distance compared to WT mice on treadmill tests (Figure S1B). There was a reduced proportion of glycolytic type IIA myofibers and increased oxidative type I myofibers in *Plk1* ^PKO^ soleus muscle (Figure S1C,D).

*Plk1* ^PKO^ mice also had significantly reduced masses of inguinal, epididymal, and anterior subcutaneous white adipose tissues (iWAT, eWAT, asWAT, respectively) (Figure S1E). iWAT adipocytes also appeared smaller in *Plk1* ^PKO^ compared to WT mice (Figure S1F). These findings together indicate that pancreatic agenesis leads to adaptive remodeling of peripheral metabolic organs.

## 4 Discussion

Our results revealed a role of PLK1 in pancreatic development. As PDX1 marks all pancreatic progenitors, *Pdx1*‐Cre should induce *Plk1* deletion in both endocrine and exocrine lineages. We observed an absence of pancreatic buds at E13.5; but how PLK1 loss‐of‐function affects earlier stages of pancreas development remains unknown. This may be addressed using *Foxa3‐Cre*, activated in endoderm of anterior hindgut region at E8.5 [9].

The pancreatic agenesis, hypoinsulinemia, and hyperglycemia of *Plk1* ^PKO^ mice resemble features of MODY [10]. Hepatic glucose production may also contribute to hyperglycemia in the *Plk1* ^PKO^ mice. Assessing gluconeogenic gene expression will address this possibility in future studies. Similar to our findings in mice, MODY patients have normal insulin sensitivity despite β‐cell dysfunction [10]. Insulin deficiency can improve systemic insulin sensitivity through adaptive changes in peripheral tissues [11, 12]. The increased proportion of type I myofibers in soleus muscles of *Plk1* ^PKO^ mice may underly the altered insulin sensitivity. These fibers have higher mitochondrial content and oxidative capacity compared to type II fibers, and are generally more insulin‐sensitive [13]. The fiber‐type switch may alternatively reflect a metabolic adaptation to insulin deficiency. It is plausible to hypothesize that MODY patients also exhibit fiber type switching toward oxidative myofibers.

The increased VO_2_ and reduced RER in *Plk1* ^PKO^ mice indicate systemic metabolic remodeling to favor lipid oxidation. The reduced adiposity in the *Plk1* ^PKO^ mice is consistent with this notion. Similarly, increased energy expenditure and substrate flexibility have been reported in diabetes patients [14]. Despite adaptation for 8 generations, survival of *Plk1* ^PKO^ mice progressively decreases over time, possibly due to loss of genetic variability caused by inbreeding [15].

Pancreas agenesis is a congenital malformation with unclear etiology. Among 53 documented cases of human dorsal pancreas agenesis, 53% had hyperglycemia [16]. Although several genes are linked to pancreas agenesis in mice [17], none is associated with human diseases. It is imperative to investigate if PLK1 mutations are linked to human pancreas agenesis.

## Author Contributions

X.C. and S.K. conceived the project. X.C. and Z.J. designed and performed the experiments. X.C. and S.K. analyzed the data and wrote the manuscript. S.K. provided financial support. S.K. is the guarantor of this work and, as such, had full access to all the data in the study and takes responsibility for the integrity of the data and the accuracy of the data analysis.

## Conflicts of Interest

The authors declare no conflicts of interest.

## Supporting information


**Figure S1:** fsb270946‐sup‐0001‐FigureS1.pdf.

## Data Availability

Data will be made available on request.
