# Supplementary material for: A Mouse Model of Partial Pancreas Agenesis Induced by Polo‐like kinase 1 Mutation
Source: FASEB J. 2025 Aug 13;39(16):e70946. doi: 10.1096/fj.202501377R (PMC12345124; doi:10.1096/fj.202501377R)
Supplement: Supplementary file 1 — Figure S1: fsb270946‐sup‐0001‐FigureS1.pdf. [file FSB2-39-e70946-s001.pdf]

Supplemental Information

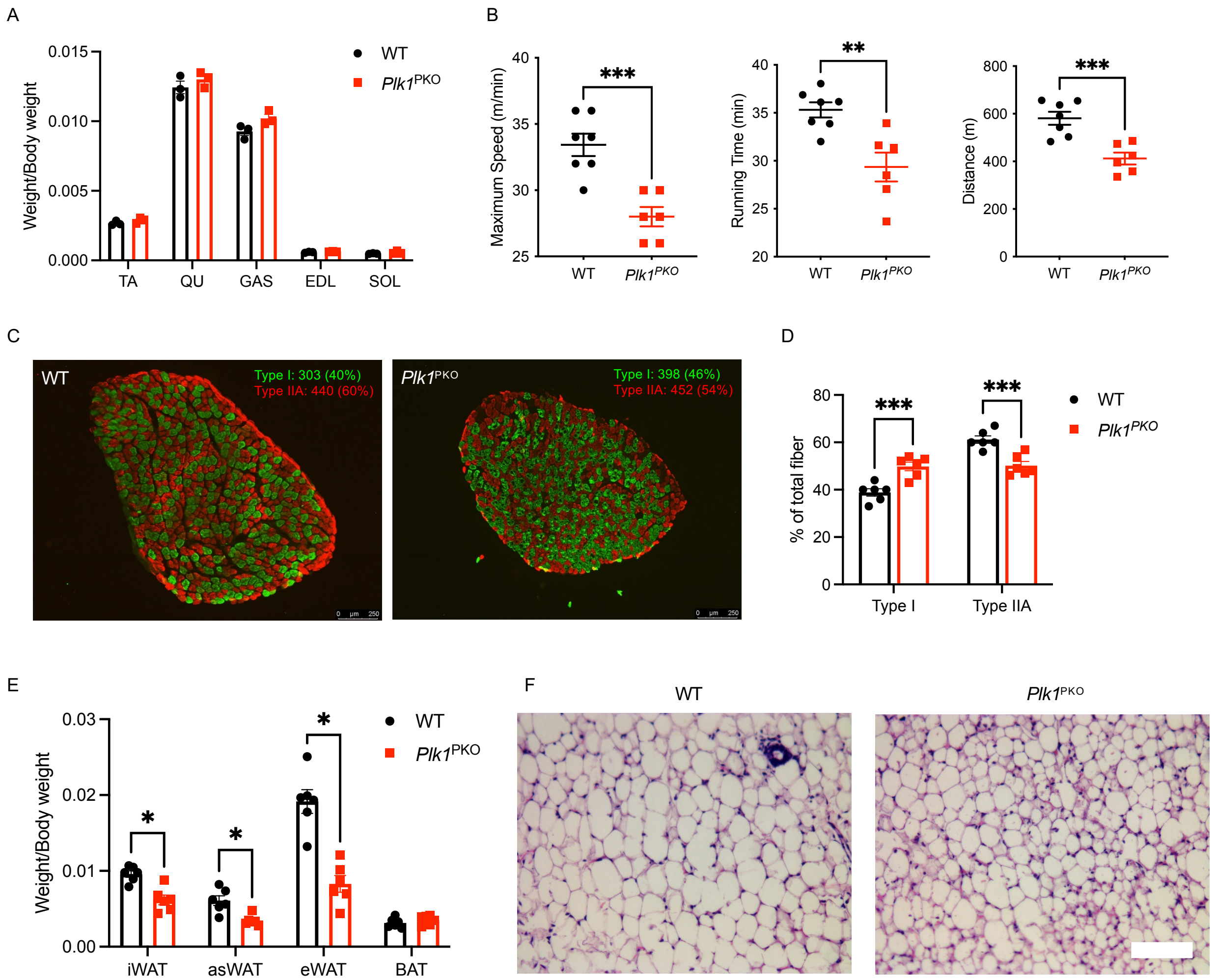

Supplemental Figure S1 *Plk1*ᴾᴷᴼ mice exhibit SOL muscle fiber type switch and reduced motor performance, and reduced WAT mass.

(A) Quantification of muscle weight/body weight from WT and *Prmt5*ᵁᴷᴼ mice at 3-month-old (n=6 mice per group).

(B) Exercise performance including maximum speed (left), running time (middle) and distance (right) of 3-month-old WT and *Plk1*ᴾᴷᴼ mice measured by treadmill (WT, n = 7 mice; *Plk1*ᴾᴷᴼ, n = 6 mice).

(C) Type I and IIA myofibers staining in SOL muscles of 3-month-old WT and *Plk1*ᴾᴷᴼ mice. Scale bar: 250 µm.

(D) Quantification of abundance of various fiber types in SOL muscles of 3-month-old WT and *Plk1*ᴾᴷᴼ mice (n=6 mice per group).

(E) Quantification of adipose tissue weight/body weight from 3-month-old WT and *Plk1*ᴾᴷᴼ mice (n=6 mice per group).

(F) Representative H&E staining of iWAT from 3-month-old WT and *Plk1*ᴾᴷᴼ mice. Scale bar: 200 µm.
